# Versican expression in canine carcinomas in benign mixed tumours: is there an association with clinical pathological factors, invasion and overall survival?

**DOI:** 10.1186/1746-6148-8-195

**Published:** 2012-10-20

**Authors:** Karine A Damasceno, Angélica C Bertagnolli, Alessandra Estrela-Lima, Lorena GR Ribeiro, Bruna S Rabelo, Cecília B Campos, André LB Barros, Geovanni D Cassali

**Affiliations:** 1 Department of General Pathology, Biological Sciences Institute, Universidade Federal de Minas Gerais, Belo Horizonte, MG, Brazil; 2 Fepagro Animal Health, Desidério Finamor Institute of Veterinary Research (IPVDF), Eldorado do Sul, RS, Brazil; 3 Department of Pathology and Clinics, School of Veterinary Medicine and Zootechny, Universidade Federal da Bahia, Salvador, BA, Brazil; 4 Department of Clinical and Toxicological Analyses, School of Pharmacy, Universidade Federal de Minas Gerais, Belo Horizonte, MG, Brazil

**Keywords:** Versican, Mixed tumour, Carcinoma, Invasion, Canine

## Abstract

**Background:**

Components of the extracellular matrix have been studied in an attempt to elucidate the mechanisms involved in the biological behaviour of tumours. The presence of the proteoglycan versican has been strongly associated with cancer development and progression. However, relationship between versican expression and clinical pathological factors and overall survival has not been previously studied in veterinary medicine. Carcinomas in benign mixed tumours (CBMTs) are one of the most common malignant tumours in female canines and can serve as models for studies of tumour progression. The aim of this study was to evaluate the expression of versican in *in situ* and invasive carcinomatous areas of canine CBMTs and to evaluate possible associations of versican expression with other classic prognostic factors and overall survival.

**Results:**

Clinical staging; histological grade determination; immunohistochemical staining for versican, E-cadherin and Ki-67; and confirmation of invasion areas by staining for p63 and smooth muscle α-actin (α-SMA) were performed on 49 canine cases of CBMT. Tumour invasion was considered when suspicious Haematoxylin-Eosin (HE)-stained areas showed a total loss of α-SMA and p63 immunoreactivity. Versican immunoreactivity was less intense in the areas adjacent to the *in situ* carcinomatous regions, compared to invasive regions, which showed extensive and strong staining.

**Conclusions:**

Our data reveal that in canine CBMTs, versican expression differs significantly between invasive and *in situ* areas, suggesting a role for this molecule in tumour progression. Although a direct relationship exists between versican and invasiveness, our results indicate that the isolated evaluation of this proteoglycan does not represent an independent prognostic factor in canine CBMTs.

## Background

Mammary neoplasms are the most common proliferative lesions among female dogs [1], representing approximately 52% of all neoplasms. The malignant ratio of these tumours is variable, with an average of 50% [2-4]. Canines between seven and eleven years of age are the most commonly affected [5-7].

Among malignant canine mammary tumours, carcinomas in benign mixed tumours (CBMTs) are the most common histological type in the authors’ experiences [3,8]. These tumours originate from a malignant transformation of the epithelial component of a benign mixed tumour. This carcinomatous proliferation may occur as *in situ* or infiltrative growth, as demonstrated either by a loss in continuity of the myoepithelial and basal layers associated with the neoplastic cells invading the stroma or by complete replacement of the pre-existing benign lesion at the time of histopathological examination [9].

Since the 1970s, authors have defended the malignant progression hypothesis in benign canine mammary mixed tumours [10]. More recently, protein alterations that may contribute to the transformation of benign mixed tumours have been observed, such as the loss of p63, ΔNp63, E-cadherin, β-catenin and EGFR overexpression [11-13].

Genetic factors that result in the malignization process are still relatively unknown. However, phenotypic evaluations of myoepithelial cells and extracellular matrix components have been performed in the attempt to clarify the mechanisms involved in the biological behaviour of these tumours [14].

Among the extracellular matrix components, the proteoglycan versican has caught the attention of researchers [15,16]. Versican is produced by stromal cells in a wide range of mature tissues, including smooth muscles, cartilage, and skin [17]. Some studies also suggest that versican is involved in cancer development and progression [15,18,19] because higher expression levels have been associated with local invasion and angiogenesis in breast cancer in women [16].

Elevated versican expression in peritumoural stromal tissues has also been associated with histological grade and may be a strong factor in predicting disease relapse in lymph node negative breast cancer patients [20]. The mechanisms that alter the expression of this proteoglycan are still poorly understood; however, its role in modulating the loss of adhesion and cell motility has also been recognised in cases of breast cancer metastasis [16,20].

Researchers have demonstrated that in canine mammary tumours, versican is highly expressed in proliferating fusiform cells and in myxoid areas of the mixed tumours [21]. Versican accumulation in myoepithelial tumours is related to the early differentiation of the myxoid matrix to cartilage. In prior studies, these same authors observed increased versican expression in areas of tumour infiltration [14].

Considering that CBMTs can serve as research models for tumour progression [13,22], the analysis of versican expression in these tumours can contribute to the understanding of the transformation and progression mechanisms in malignant mammary tumours. In this context, the present work aims to evaluate the expression of the proteoglycan versican in *in situ* and invasive carcinomatous areas in canine CBMTs and to verify its association with other prognostic factors and overall survival.

## Results

### Versican expression in peritumoural stroma

**Figure 1:**
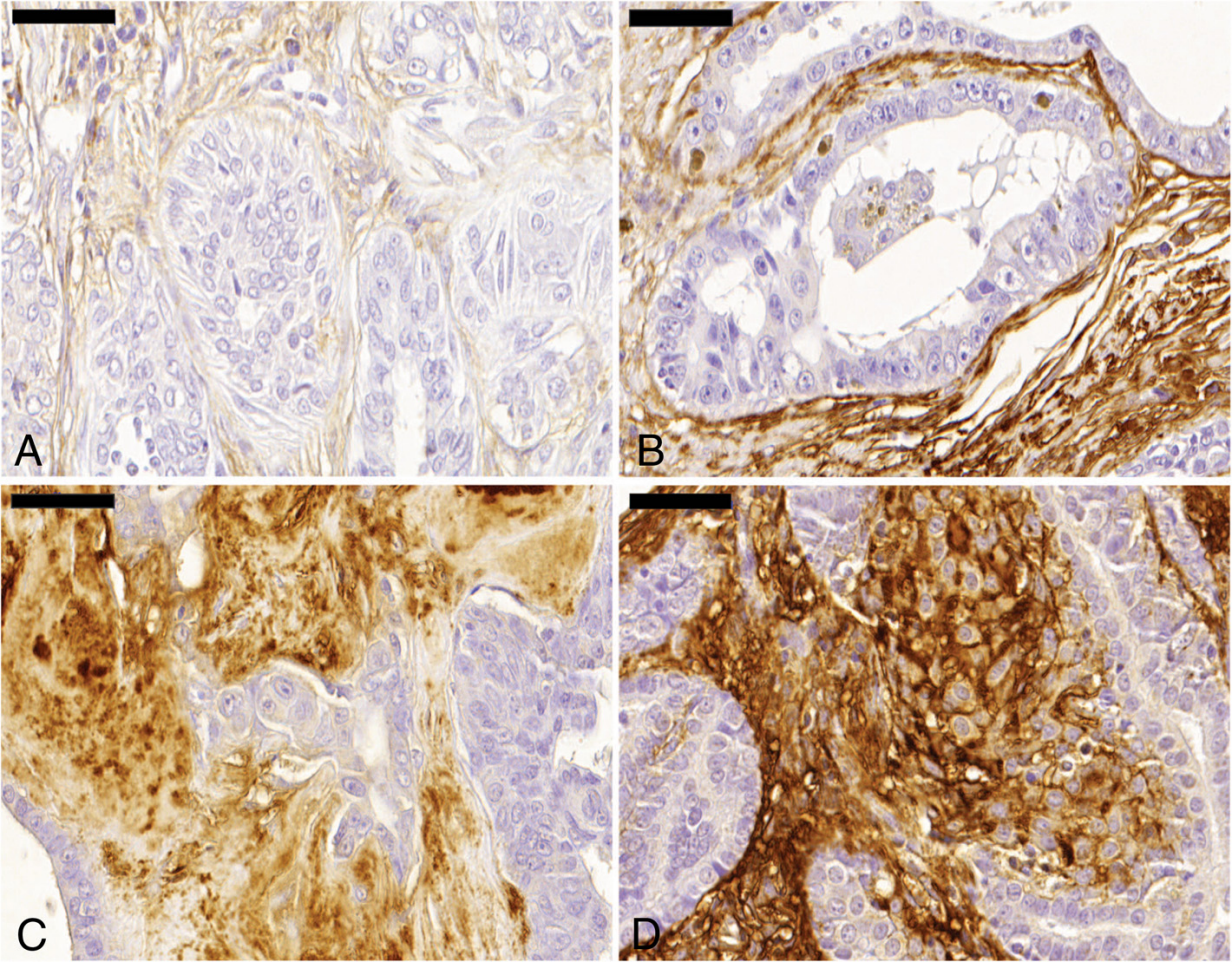
**Carcinoma in benign mixed tumour.A**. *In situ* carcinomatous area with low stromal versican expression. Immunohistochemical stain with Mayer’s haematoxylin counterstain, 40×. **B**. *In situ* carcinomatous area with moderate stromal versican expression. Immunohistochemical stain with Mayer’s haematoxylin counterstain, 40×. **C**. Versican moderate expression adjacent to invasive area. Immunohistochemical stain with Mayer’s haematoxylin counterstain*,* 40×. **D**. Versican overexpression adjacent to invasive area. Immunohistochemical stain with Mayer’s haematoxylin counterstain*,* 40×.

**Figure 2:**
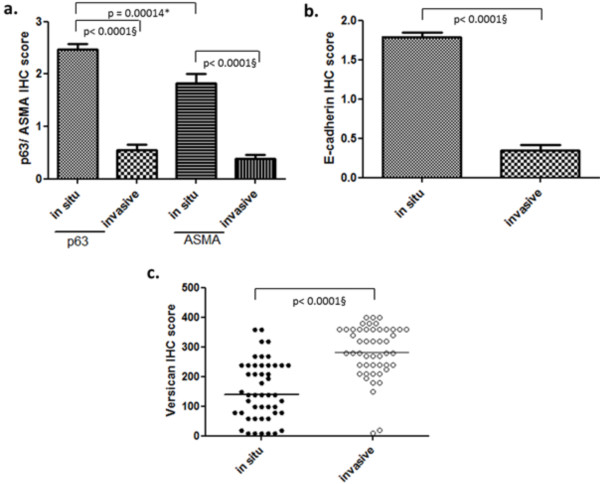
**Immunoreactivity for p63, α-SMA, E-cadherin and Ki-67 in *in situ *and invasive areas.** Difference between *in situ* and invasive areas for p63 and α-SMA (**a**) and e-cadherin (**b**) demonstrated by immunohistochemistry. Accumulation of versican in invasive carcinomatous areas when compared to *in situ* areas (**c**). *Spearman correlation. §Wilcoxon test.

Proteoglycan versican immunoreactivity in areas adjacent to the *in situ* carcinomatous regions were less intense (median, 140.0) compared to the areas adjacent to the invasive regions (median, 280.0), which were characterized by more extensive areas of strong versican expression (Figures 1 and 2).

Table 1 presents the results of the versican analysis and shows the intensity and percentage of the stained areas in both the *in situ* and invasive regions.

**Table 1:** Versican immunohistochemistry evaluation in mammary carcinomas in benign mixed tumours in female dogs

***Areas***	***Parameters***	***All***	***Score***	***Group 1 (n = 25)***	***Group 2 (n = 24)***	***P value***
***In situ***	*Intensity*	49	1	6/25	1/24	0.0002
			2	15/25	5/24	
			3	4/25	14/24	
			4	0/25	4/24	
	*Percentage of the tissue section stained positive (mean)*	49		43.24	68.96	
	*Intensity*	49	1	1/25	0/24	*<0.0001*
			2	1/25	0/24	
			3	17/25	0/24	
			4	6/25	24/24	
	*Percentage of the tissue section stained positive (mean)*	49		67.6	88.96	
***P value***				*<0.0001*	*<0.0001*	

Group 1, represented by cases with low versican expression; and Group 2, represented by cases with versican overexpression.

Wilcoxon Test.

### Clinical and pathological features

The present study demonstrated a higher incidence of the disease among Poodles (55.77%), followed by mongrel dogs (9.62%) and Doberman Pinschers (9.62%). The age of the dogs varied from 4.5 to 19 years, with a mean age of 10.5 years. Comparative analyses of the clinical and pathological characteristics among these groups are presented in Table 2.

**Table 2:** Association between pathological and clinical status of mammary carcinomas in mixed tumours in female dogs

***Parameters***	***All (n = 49)***	***Group 1 (n = 25)***	***Group 2 (n = 24)***	***P value***
***Lymph node metastasis***	6/49	3/25	3/24	-
***Pulmonary metastasis***	1/49	0/25	1/24	-
***Size***				
*< 3 cm*	28/49	13/25	15/24	
*3 < x < 5*	11/49	6/25	5/24	*0.3735*
*≥ 5 cm*	10/49	6/25	4/24	
***Clinical staging***				
*I*	24/49	12/25	12/24	
*II*	11/49	6/25	5/24	
*III*	7/49	4/25	3/24	*0.9657*
*IV*	6/49	3/25	3/24	
*V*	1/49	0/25	1/24	
***Histological grade***				*0.714*
*I*	44/49	21/25	23/24	
*II*	5/49	4/25	1/24	
*III*	0/49	0/25	0/24	

Group 1, represented by cases with low versican expression; and Group 2, represented by cases with versican overexpression.

Student’s t- test was used for parametric data (size).

Fisher’s exact test used for categorical variables (clinical staging and histological grade).

### Invasion, loss of adhesion, and proliferation index

**Figure 3:**
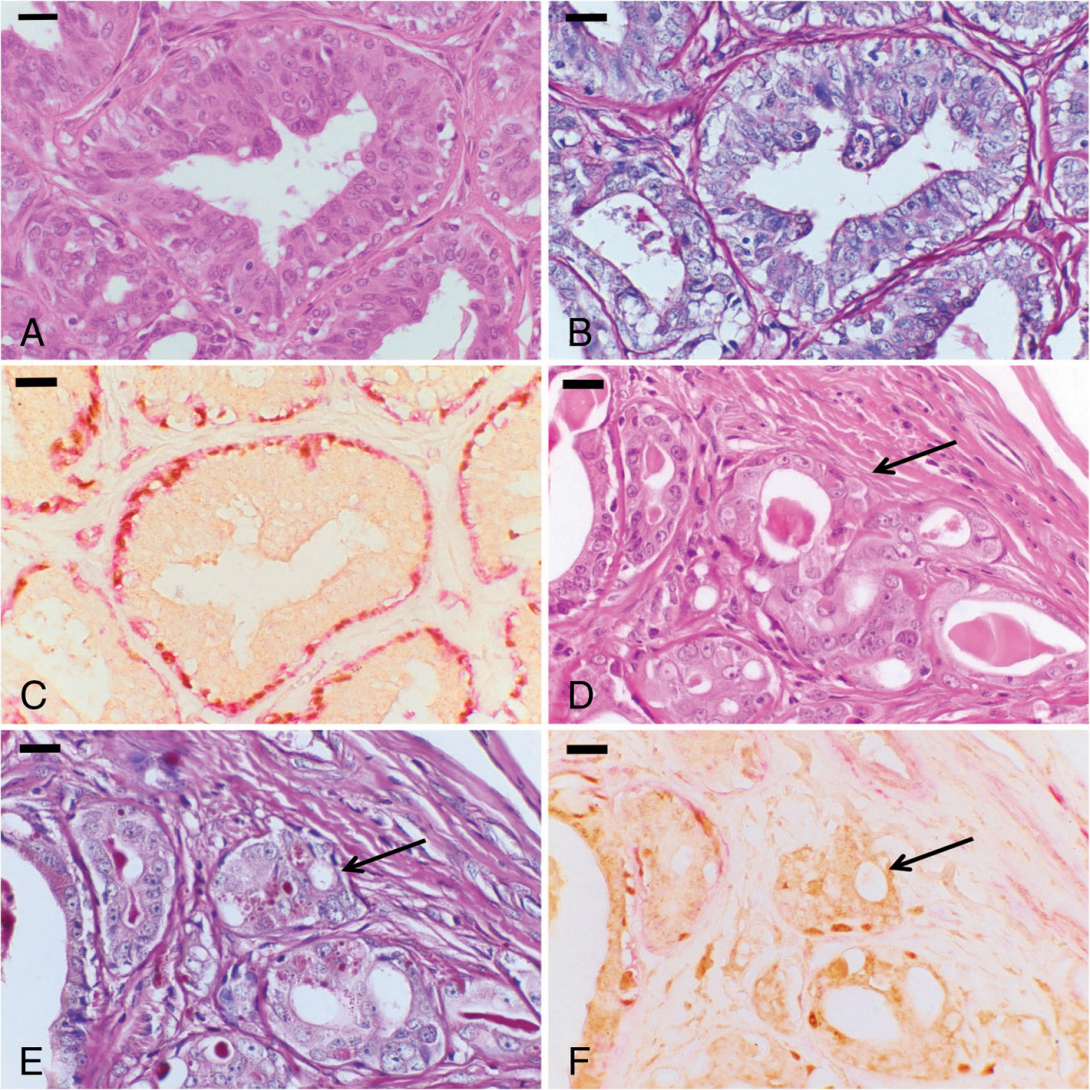
**Carcinoma in benign mixed tumour.A**. *In situ* carcinomatous area in a carcinoma in benign mixed tumour. HE, 60×. **B**. Evidence of an intact basement membrane. PAS, 60×. **C**. Integrity of the myoepithelium layer demonstrated by p63 and α-SMA double staining. 60×. **D**. Microinvasion area in carcinoma in benign mixed tumour (arrow). HE, 60×. **E**. Discontinuous basement membrane (arrow). PAS, 60×. **F**. Absence of p63 and α-SMA expression (arrow), 60×.

The evaluation of the myoepithelial cell layer integrity was performed through analysis of p63 and α-SMA immunoreactivity (Figure 3). The *in situ* areas were defined through the observation of epithelial cells that were in a tubular arrangement with basal membrane integrity shown by HE staining and cells that were double-positive for p63 and α-SMA.

Invasion was considered when suspicious HE-stained areas revealed a total loss of immunoreactivity for α-SMA and p63, thus denoting a loss of myoepithelial cells in gaps corresponding to a three-cell space [23]. These results are represented in Table 3.

**Table 3:** Evaluation for PAS and association with immunoreactivity for p63, α-SMA, E-cadherin and Ki-67 in groups

	***Score***	***Areas***			
		***In situ***		***Invasive***	
		***Group 1***	***Group 2***	***Group 1***	***Group 2***
***p63***	***0***	0/20	1/21	12/21	13/21
	***1***	0/20	3/21	6/21	5/21
	***2***	7/20	6/21	3/21	3/21
	***3***	13/20	11/21	0/21	0/21
α***-SMA***	***0***	1/20	6/21	13/21	14/21
	***1***	5/20	5/21	8/21	6/21
	***2***	4/20	3/21	0/21	1/21
	***3***	10/20	7/21	0/21	0/21
***E-cadherin***	***0***	0/24	0/24	17/25	15/24
	***1***	3/24	7/24	8/25	9/24
	***2***	21/24	17/24	0/25	0/24
***Ki-67 (Mean value)***		5.6 (22)	8.7 (22)	5.62 (22)	12 (22)

Group 1, represented by cases with low versican expression; and Group 2, represented by cases with versican overexpression.

Significant differences at *P* < 0.0001 between *in situ* and invasive areas in p63, α-SMA, and e-cadherin using the Wilcoxon Test.

Rupture of the basal membrane, which is associated with the observation of malignant epithelial cells in the adjacent stroma, was also observed by PAS staining (Figure 3).

Evaluation of p63 and α-SMA staining in carcinomas with invasion areas showed that there was a loss of, or weak staining for, at least one marker in the remaining myoepithelial cells, which differs from observations in *in situ* areas, in which double-positive staining was notable. A significant difference was observed in p63 and α-SMA expression between *in situ* and invasive areas, with *P* < 0.0001 (Figure 2).

**Figure 4:**
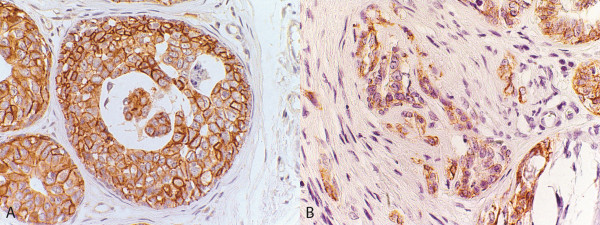
**Carcinoma in benign mixed tumour.A**. *In situ* carcinomatous area with high E-cadherin expression in cell membrane. Immunohistochemical stain with Mayer’s haematoxylin counterstain, 40×. **B**. Loss of E-cadherin expression in invasive carcinomatous cells. Immunohistochemical stain with Mayer’s haematoxylin counterstain*,* 40×.

E-cadherin, an adhesion molecule, expression in *in situ* and invasive areas (Figure 4) was evaluated to investigate its possible relationship with versican. *In situ* carcinomatous areas revealed strong and diffuse immunoreactivity for E-cadherin, whereas invasive areas showed weak or absent staining. In tumours with low (group 1) and high (group 2) versican expression, a significant difference in the expression of E-cadherin was observed between the *in situ* and invasive areas, with *P* < 0.0001 (Figure 2). However, no statistically significant correlation could be observed between versican and E-cadherin expression.

### Survival curves

**Figure 5:**
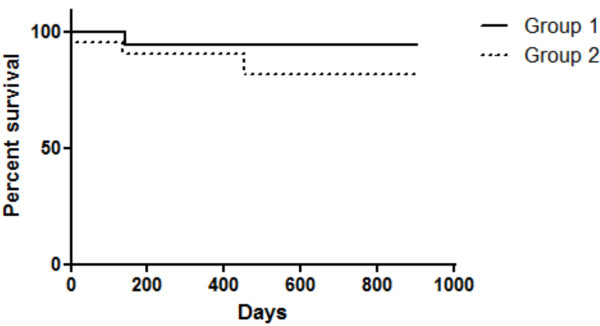
**Survival rates of animals with canine mammary carcinomas in benign mixed tumours.** Survival curves were estimated with the Kaplan-Meier method followed by the log-rank test. Group 1, represented by cases with low versican expression; and Group 2, represented by cases with versican overexpression.

Considering only versican expression, overall survival was found to be longer in female dogs with low immunostaining for this proteoglycan than in dogs with high immunostaining; however, no statistically significant difference was observed (*P* = 0.1947) (Figure 5).

Overall survival was analysed in 40 cases. Thirty-two (80%) dogs remained alive at the end of the study, four (10%) dogs died during the study and four (10%) were censored due to loss of follow-up or death unrelated to the tumour.

## Discussion

The proteoglycan versican is one of the most studied components of the extracellular matrix associated with human breast cancer [24]. Versican is synthesized mainly by stromal cells and possesses anti-adhesive properties that interfere with cell motility [25]. This property appears to be related to the association of versican with hyaluronic acid, which exhibits altered expression in neoplastic conditions [26].

The most important criteria for the diagnosis of invasion in mammary carcinomas is the absence of myoepithelial cells surrounding neoplastic proliferations associated with basal layer rupture [23]. Nevertheless, the recognition of these areas based solely on HE staining evaluation can be quite difficult. Therefore, staining for p63, α-SMA and PAS was implemented to aid in the evaluation of invasive regions.

In this study, myoepithelial cells revealed a progressive loss of immunoreactivity for both p63 and α-SMA from *in situ* to invasive areas. In *in situ* areas, the aberrant expression of these molecules suggests that myoepithelial cells have suffered dedifferentiation and no longer express important functional and characterization molecules [12,27,28]. In sites suspected of invasion, the absence of p63 and α-SMA staining denotes a flaw in the myoepithelial layer, which in association with the discontinuity of the basal membrane, confirms the lesion. In such sites, double-staining immunohistochemistry is useful for evaluating these areas.

Once areas with infiltration of neoplastic cells in the adjacent stroma were confirmed, the presence of versican was evaluated. Versican expression differed significantly between *in situ* and invasive areas (*P* < 0.0001), both in cases of low and of high versican expression. There was also statistically significant difference (*P* = 0.0002) in the versican immunoreactivity in the *in situ* areas between the two groups (1 and 2). In addition, a significant difference was observed when comparing versican immunoreactivity in invasive and *in situ* areas (*P* < 0.0001), suggesting a direct relationship between versican and invasion.

Researchers have previously analysed versican expression in areas adjacent to *in situ* and invasive carcinomatous areas in human breast cancer [24]. The mechanisms involved in cell and stromal interaction are believed to lead to the development of invasive lesions from *in situ* mammary lesions. Furthermore, elements typically expressed in the invasive stroma, such as versican, suggest that pre-invasive lesions can acquire some characteristics commonly attributed to invasive areas, most likely contributing to the progression of neoplastic processes.

Many authors have also shown that extracellular matrix components, including the proteoglycan versican, play a critical role in facilitating the progression and dissemination of malignant neoplastic cells [14,18,19,26,29-31]. Mukaratirwa and colleagues (2004) observed a significant correlation between stromal immunoreactivity intensity for versican and invasion in colorectal carcinomas in dogs, suggesting that this proteoglycan supports tumour progression [26]. The carcinomatous cells that invade the stroma are believed to be capable of stimulating fibroblasts to produce versican, which in turn plays a crucial role in tumour progression.

In this study, the possible association between versican expression and clinico-pathological factors was evaluated to verify the biological significance and prognostic value of versican. However, no statistically significant relationship was observed between the evaluated prognostic factors and high or low expression of versican or between the prognostic factors and versican staining in *in situ* and invasive areas. These results may be explained by the fact that the studied cases mostly represented low grade, nonaggressive tumours associated with a good prognosis, suggesting that our samples were somewhat homogenous. Furthermore, no association was found between the evaluated biological factors and the immunohistochemical expression of versican in human pharyngeal squamous cell carcinomas [15]. In non-small cell lung cancer, versican has been shown to be associated with an unfavourable prognosis but is not considered to be an independent indicator of patient survival [32]. Finally, in ovarian cancer, versican expression does not appear to have prognostic significance, despite the finding that high stromal staining is related to poor disease-free survival [33].

Versican has also been linked to neoplastic cell adhesion and proliferation [15,16,21,34-37]. A potential significant role for the G3 versican domain, with its EGF-like motif, in influencing tumour cell viability, proliferation and local tumour growth has been suggested [16]. Some studies also suggest that versican expression is involved in an increase in cell proliferation, apoptosis-resistance and the regulation of the cadherin family protein expression, inducing mesenchymal-epithelial transitions [38,39]. Versican isoforms v1 and v2 seem to play distinct roles in cell proliferation functions that are mediated by the GAG-α and GAG-β domains, respectively [34,38]. It has been shown that the GAGβ domain is responsible for activation, whereas the GAGα domain plays a role in the suppression of epidermal growth factor receptor (EGFR) expression and its downstream signaling pathway [38]. The extracellular environment might become favourable for cell proliferation and survival when v1 expression is increased, as in the case of tissue development and tumour formation [38].

In canine mammary tumours, the mechanisms that regulate the signal pathways and the role of the different isoforms remain poorly elucidated. In this study, no difference regarding the proliferation index was observed between low and high versican expression groups. Thus, Ki-67 expression did not indicate a relationship between proteoglycan overexpression and tumour proliferation in CBMTs.

Studies indicating a relationship between versican and E-cadherin expression in epithelial tumours are scarce. Therefore, one goal of this study was to investigate the association between versican and E-cadherin. However, despite the existence of statistically significant differences between both E-cadherin and versican expression in *in situ* and invasive areas, no relationship between the two molecules was observed in CBMTs. This finding most likely indicates that versican expression interferes in carcinomatous cell adhesion through other mechanisms that are not yet understood.

## Conclusions

Our data reveal that in CBMTs, versican expression differs significantly between invasive and *in situ* areas, suggesting a role for this molecule in tumour progression. However, no associations between the proteoglycan and prognostic factors could be observed. Therefore, although a direct relationship exists between versican expression and invasiveness, our results indicate that isolated evaluation of this proteoglycan does not represent an independent prognostic factor for canine CBMTs.

## Methods

### Case selection

To carry out this study, 49 cases of CBMTs were selected at the Comparative Pathology (Universidade Federal de Minas Gerais) and Pathological Anatomy Laboratories (Universidade Federal da Bahia). Samples of mammary tumours were obtained from female dogs, regardless of breed or age, that had undergone mastectomy. Clinico-pathological parameters, including tumour size, lymph node metastasis, pulmonary metastasis, clinical stage and histological grade were used in comparative analyses between low and high versican expression groups. According to the a median expression of versican in invasive areas, two distinct categories were determined: Group 1, represented by cases with values of versican expression that were lower than the median; and Group 2, represented by cases with values of versican expression that were higher than the median.

### Clinical staging: TNM

Clinical staging was based on tumour size (T), regional lymph node involvement (N) and presence of distant metastasis (M) and was determined according to the TNM staging system, established by the World Health Organization (WHO) for canine mammary tumours [40]. Data were obtained from a retrospective review of the clinical, radiological, and pathological records of all animals.

### Histological classification and grading

Fragments of the affected mammary glands that included skin and subcutaneous tissues were fixed in phosphate-buffered 10% neutral formalin and processed by the routine technique of paraffin embedding. Histological sections of 4 μm were obtained from the tumour samples and stained using HE [41]. In all cases, duplicate slides were prepared and analysed by two veterinary pathologists, and the confirmation of the histological type followed the standards proposed by Misdorp et al. (1999) [42].

Histological analyses of 49 lymph nodes were further performed to categorise tumour samples with or without lymph node metastasis.

The tumours were graded by the Nottingham System [43], and the criteria included tubular formation, nuclear pleomorphism and mitotic index. Mitotic activity was determined as the number of mitotic cells per 10 fields, performed by two independent analysts in a blinded fashion, using an Olympus BX-40 microscope fitted to a 10× eye piece and a 40× objective. Using this equipment, one high-power field visualises an area of 0.239 mm2 [44].

### Immunohistochemistry

The following monoclonal antibodies were used in the present study: versican (12C5, DSHB, 1:50), p63 (4A 4, Neomarkers, 1:80), smooth muscle α-actin (α-SMA) (1A 4, Dako, 1:100), E-cadherin (NCH-38, Dako, 1:60) and ki-67 (Mib-1, Dako, 1:25). Three micrometre tissue sections were cut from one representative block of each case and collected onto glass slides. Tissue sections were deparaffinised in xylene, subjected to heat-induced antigen retrieval with an antigen retrieval solution (DAKO) at pH 6.0 in a water bath at 98°C for 20 min, and then incubated for 60 minutes at room temperature with the monoclonal antibodies (all except versican). For versican antigen retrieval, chondroitinase ABC (from Proteus vulgaris; Sigma Chemicals) digestion was performed at 37°C for 90 min with 0.5 U/mL of the enzyme in 0.25 M Tris buffer (pH 8.0) containing 0.18 M sodium chloride and 0.05% bovine serum albumin (BSA). Next, 0.25 M Tris buffer (pH 8.0) containing 0.1 M 6-amino-n-caproic-acid and 5 mM benzamidine hydrochloride was used to inhibit protease activity for 30 min [21]. Slides were incubated overnight at 4°C with the monoclonal antibody 12C5. An endogenous peroxidase activity block was performed with 3% hydrogen peroxidase in methanol. A biotin-peroxidase system was then used in the immunohistochemical procedure, with identification of the secondary antibody using a polymer (ADVANCE HRP–ready to use–DakoCytomation). Diaminobenzidine was used as a chromogen, and the sections were counterstained with Mayer’s haematoxylin, dehydrated, and mounted in synthetic medium. The EnVision™ G|2 Doublestain System Kit, Rabbit/Mouse (DAB+/Permanent Red) was used for the double-staining immunohistochemical technique (used to identify p63/ α-SMA).

Negative controls were obtained by omitting the primary antibodies. Adjacent normal canine mammary tissues were used as positive controls for p63, α-SMA and E-cadherin. Canine mammary tumours previously known to express high levels of Ki-67 and abundant myxoid tissues were used as positive controls for Ki-67 and versican, respectively.

### Immunohistochemical evaluation

Areas previously suspected of stromal invasion by HE staining were confirmed through the presence of both p63 and α-SMA. Immunoreactivity for p63 and α-SMA was assessed semiquantitatively using a scoring system: (−) no staining, (+) weak or <5% of stained myoepithelial cells, (++) moderate or between 5 and 50% stained myoepithelial cells, and (+++) strong or 50% of stained myoepithelial cells [12].

*In situ* and invasive carcinomatous areas of CBMTs were analysed in at least five fields in this study. In these areas, versican expression was assessed by semiquantitative scoring [31] that includes (i) the overall percentage of the tissue section stained positive (0-100%), and (ii) the signal intensity (4-point scale) for each proteoglycan. The scoring used for the 4-point scale was as follows: 1, negative or very weak staining; 2, weak positive; 3, moderate positive; and 4, strong positive. The semiquantitative immunohistochemical (IHC) score for the expression level of each proteoglycan was provided by the multiplication of the percentage (0–100) of the tissue section staining positive by the factor (1–4) corresponding to the staining intensity of the tissue section. According to the final results of this evaluation, a median versican expression score (280.0) was obtained for invasive areas. Thus, two distinct categories were determined: Group 1, represented by cases with values of versican expression considered lower than the median, and Group 2, represented by cases with values of versican expression that were higher than the median.

Ki-67 staining was considered positive when cell nuclei presented a diffuse nuclear staining pattern. Ki-67 proliferative activity was assessed with an image analysis determining the percentage of positive cells among 1000 tumour cells (proliferation index) [44].

E-cadherin staining of the membranes of neoplastic epithelial cells was evaluated in *in situ* and invasive areas. Immunostaining intensity for E-cadherin was evaluated qualitatively and scored as follows: 0 = negative, when the staining was absent; 1 = moderate, when the staining was weaker than in normal epithelium; and 2 = strong, when the staining was equal to that observed in normal mammary epithelium [45].

### Histochemistry

Periodic Acid Schiff (PAS) staining was performed on all sections. This reaction indicates the presence of polysaccharides in the basal membrane and verifies the integrity of this structure [46].

### Statistical analysis

To determine whether continuous variables (size, age, Ki-67) differed between groups with low and high versican expression, Student’s *t*-test was used for parametric data, and the Mann–Whitney Test was used for non-parametric data. For categorical variables, such as lymph node metastasis, clinical staging, mitotic index, histological grade, and α-SMA and p63 expression, Fisher’s exact test was performed.

Differences in versican, Ki-67, p63, α-SMA and E-cadherin expression between *in situ* and invasive areas were evaluated by the Wilcoxon Test.

Kaplan Meyer analysis (log-rank test) was used to evaluate overall survival. Values were considered statistically significant when *P* < 0.05. Overall survival was defined (in days) as the period between surgical excision of the primary tumour and the death of the animal due to the disease. Animals with loss of follow-up or death unrelated to the tumour were censored.

Clinical follow-up of the 40 animals included in the overall survival analysis was performed from 2008 to 2011 through periodic telephone communication and return visits to the Veterinary Teaching Hospital of the Federal University of Minas Gerais. Overall survival time was defined as the period (in days) between the date of surgical removal of the tumour and death caused by the disease. Animals that died from unknown causes or causes unrelated to the tumour were censored.

### Ethic aspects

All procedures were performed under the guidelines and with the approval of the Ethics Committee in Animal Experimentation (CETEA/UFMG), protocol 219/2009.

## Abbreviations

CBMT: Carcinoma in benign mixed tumour; α-SMA: Smooth muscle α-actin; HE: Haematoxylin-Eosin; EGFR: Epidermal growth factor receptor.

## Competing interests

The authors declare that they have no competing interests.

## Authors’ contributions

KAD: conceived the study, performed immunohistochemical techniques and statistical analyses, analysed data and drafted the manuscript. ACB: assisted in the immunohistochemical analyses, performed the statistical analyses and participated in the design of the study. AEL: participated in the design of the study, provided cases for the study and revised the manuscript. LGRR: collected cases and clinical data and revised the manuscript. BSR: participated in the immunohistochemistry and analysed data. CBC: collected clinical data and revised the manuscript. ALBB: assisted in standardising the immunohistochemical technique. GDC: participated in the study design, performed histological and immunohistochemical analyses, coordinated the study and revised the manuscript. All authors read and approved the final manuscript.
